# Solid-Phase Synthesis of Difficult Purine-Rich PNAs through Selective Hmb Incorporation: Application to the Total Synthesis of Cell Penetrating Peptide-PNAs

**DOI:** 10.3389/fchem.2017.00081

**Published:** 2017-10-17

**Authors:** Julien Tailhades, Hotake Takizawa, Michael J. Gait, Don A. Wellings, John D. Wade, Yoshitsugu Aoki, Fazel Shabanpoor

**Affiliations:** ^1^The Florey Institute of Neuroscience and Mental Health, University of Melbourne, Parkville, VIC, Australia; ^2^Department of Biochemistry and Molecular Biology, Monash University, Clayton, VIC, Australia; ^3^Department of Molecular Therapy, National Center of Neurology and Psychiatry, Tokyo, Japan; ^4^Laboratory of Molecular Biology, Medical Research Council, Cambridge, United Kingdom; ^5^SpheriTech Ltd., Runcorn, United Kingdom; ^6^School of Chemistry, University of Melbourne, Parkville, VIC, Australia

**Keywords:** duchenne muscular dystrophy, peptide nucleic acid, cell penetrating peptide, Hmb incorporation, PNA conjugation

## Abstract

Antisense oligonucleotide (ASO)-based drug development is gaining significant momentum following the recent FDA approval of Eteplirsen (an ASO based on phosphorodiamidate morpholino) and Spinraza (2′-*O*-methoxyethyl-phosphorothioate) in late 2016. Their attractiveness is mainly due to the backbone modifications which have improved the *in vivo* characteristics of oligonucleotide drugs. Another class of ASO, based on peptide nucleic acid (PNA) chemistry, is also gaining popularity as a platform for development of gene-specific therapy for various disorders. However, the chemical synthesis of long PNAs, which are more target-specific, remains an ongoing challenge. Most of the reported methodology for the solid-phase synthesis of PNA suffer from poor coupling efficiency which limits production to short PNA sequences of less than 15 residues. Here, we have studied the effect of backbone modifications with Hmb (2-hydroxy-4-methoxybenzyl) and Dmb (2,4-dimethoxybenzyl) to ameliorate difficult couplings and reduce “on-resin” aggregation. We firstly synthesized a library of PNA dimers incorporating either Hmb or Dmb and identified that Hmb is superior to Dmb in terms of its ease of removal. Subsequently, we used Hmb backbone modification to synthesize a 22-mer purine-rich PNA, targeting dystrophin RNA splicing, which could not be synthesized by standard coupling methodology. Hmb backbone modification allowed this difficult PNA to be synthesized as well as to be continued to include a cell-penetrating peptide on the same solid support. This approach provides a novel and straightforward strategy for facile solid-phase synthesis of difficult purine-rich PNA sequences.

## Introduction

Peptide nucleic acids (PNAs) are synthetic single-stranded analogs of DNA/RNA in which the phosphodiester backbone is replaced with a flexible and uncharged *N*-(2-aminoethyl) glycine unit and the nucleobases are linked via a methylene carbonyl linkage (Nielsen et al., 1991). This class of synthetic molecule has unique physiochemical properties including chemical stability and high bio-stability toward nucleases and peptidases in biological fluids (Demidov et al., 1994). PNAs can hybridize complimentary DNA and RNA with higher affinity and specificity than DNA–DNA or DNA–RNA duplexes (Egholm et al., 1993; Giesen et al., 1998). Since the development of PNA in 1991, it has found a wide range of chemical and biological applications (Gupta et al., 2017). One exciting application that has attracted great attention is as antisense agents in developing gene-specific therapeutics for various diseases including cancer (Alagpulinsa et al., 2015), neuromuscular diseases (Saleh et al., 2010; Yin et al., 2010; Brolin et al., 2015), as antivirals (Zeng et al., 2016), and as novel antibiotics targeting various strains of bacteria (Good and Nielsen, 1998; Pelc et al., 2015). PNAs are also being used as molecular probes for fluorescent *in situ* hybridization (FISH) (Machado et al., 2013), imaging or as biosensors in diagnostics (Tsao et al., 2014; Lee et al., 2015), and also as radiopharmaceuticals for imaging of gene expression in cancers (Suzuki et al., 2004). The PNA exerts its antisense effect through a steric blocking mechanism targeting mRNA to prevent translation or pre-mRNA to modulate the splicing to produce a therapeutically relevant mRNA transcript (Jarver et al., 2014).

Despite the widespread application of PNA, its chemical synthesis remains a challenge due to aggregation of the growing PNA chain on the solid support, particularly of purine-rich sequences. Additionally, a major side-reaction that occurs during PNA synthesis is *N*-acyl transfer of the nucleobases promoted by the nucleophilic free terminal amine during the piperidine deprotection. This problem has been addressed by using benzothiazole-2-sulfonyl (Bts) as a protecting group for the amine of PNA backbone (Lee et al., 2007). However, this does not alleviate the aggregation problem and steric hindrance during coupling of the bulky nucleobases such as guanine and adenosine. This has made it difficult to synthesize longer PNA sequences and the majority of reported studies have used short PNA of 10–15 bases in length. Although short PNA can be effective, they are prone to off-target effects which has limited their biological application. Longer PNAs (20–25 mers) can provide higher selectivity toward its target sequence (Yin et al., 2010). An improved ability to more readily synthesize longer PNA with higher purity and yields will also allow large-scale synthesis.

Some of the problems associated with PNA synthesis such as aggregation and low coupling efficiency are also present in the solid-phase synthesis of so-called “difficult peptides.” Various approaches have been developed to overcome these problems including the use of *O*-allyl-protected thymine (Altenbrunn and Seitz, 2008), pseudoprolines (Mutter et al., 2004), iso-dipeptides (Sohma et al., 2004), Hmb (2-hydroxy-4-methoxybenzyl)/Dmb (2,4-dimethoxybenzyl) backbone modifications (Offer et al., 1996; Miranda et al., 2000; Abdel-Aal et al., 2014), microwave-assisted synthesis (Pedersen et al., 2012), PEGylated solid phase matrices (Garcia-Martin et al., 2006), or ligation strategies (Kent, 2009). Some of these approaches such as the use of different matrices, coupling reagents, and Fmoc-deprotection conditions have already been tried in synthesis of PNA (Joshi et al., 2011; Pipkorn et al., 2013). These showed some improvement in the quality of synthesized PNAs which were all 10–15 mers, however, there were still significant amounts of impurities in these reported studies.

In our study, we decided to combine the best reported PNA elongation conditions as mentioned above to use Hmb or Dmb backbone modifications to overcome the effects of aggregation and increase coupling efficiency of the nucleobases during PNA assembly. To our knowledge, this is the first report on solid phase PNA synthesis that addresses synthetic difficulties from an aggregation point of view. We selected a 22-mer PNA sequence that binds to dystrophin pre-mRNA and leads to skipping of exon-8 during splicing. This skipping of exon-8 restores functional dystrophin in primary myoblast cells derived from the canine X-linked muscular dystrophy (CXMD) dog model (Yokota et al., 2009). This sequence has been used in our laboratory and by others and was chosen as a model sequence as it is difficult to synthesize in high yield and purity due to both its length and purine base content (~60%).

## Materials and methods

### Materials

Fmoc-PNA monomers were purchased from Panagene (Daejeon, Korea), Fmoc-L-amino acids, HATU, and hydroxybenzotriazole were from GL Biochem (Shanghai, China). Fmoc-TentaGel XV RAM (0.27 mmol/g, RAPP Polymer) was from Rapp Polymere (Tübingen, Germany). Piperidine, dimethylformamide (DMF), *N,N*-diisopropylethylamine (DIEA), and acetic acid (AcOH)were from Merck (Melbourne, Australia). 2,5-lutidine, acetic anhydride (Ac_2_O), 3-maleimidopropionic acid, *N,N*′-diisopropylcarbodiimide (DIC), triisopropylsilane (TIS), PBS, 2-hydroxy-4-methoxybenzaldehyde (Hmb-CHO), 2,4-dimethoxybenzaldehyde (Dmb-CHO), Sodium cyanoborohydride (NaBH_3_CN), and NH_4_HCO_3_ were obtained from Sigma-Aldrich (Castle Hill, Australia). Trifluoroacetic acid (TFA) was from AusPep (Melbourne, Australia).

### Solid-phase synthesis of peptide and PNA

PNA chain assembly was carried out on a rotary shaker N-500 manual peptide synthesizer (Kokusan Chemical, Tokyo, Japan) using TentaGel XV RAM resin at 5 μmol scale. Fmoc removal was carried out with a 20% piperidine solution in DMF for 5 min. Couplings were achieved using 3-fold excess of Fmoc-PNA monomer or Fmoc-L-amino acid activated with HATU (2.9 eq), DIEA (3 eq), and 2,5-lutidine (3 eq). The couplings were carried out at room temperature for 30 min. After each coupling steps, the capping of unreacted amino function was achieved using a solution of Ac_2_O/lutidine/DMF (v/v/v; 5/6/89) for 5 min. Following completion of the 22-mer PNA, the resin was divided into two and one half was used to continue the synthesis of a cell penetrating peptide (ApoE: LRKLRKRLLR). The second half was acylated at the N-terminus with 3′-maleimidopropionic acid (3 eq) activated with HATU (2.9 eq) in the presence of DIEA (6 eq). Side-chain protecting groups and PNA/peptide were cleaved from the resin with a solution of TFA/TIS/H_2_O (95/2.5/2.5, v/v/v) for 2 h at room temperature. After cleavage, the resin was removed by filtration, the filtrate was concentrated under a stream of nitrogen and the PNA product was precipitated in ice cold Et_2_O and washed by centrifugation three times.

### Hmb/Dmb incorporation

After Fmoc removal, the 2-hydroxy-4-methoxybenzaldehyde (Hmb-CHO, 5 eq) or the 2,4-dimethoxybenzaldehyde (Dmb-CHO, 5 eq) was dissolved in a solution of 1% AcOH in DMF, added to the resin to form the Schiff base and the reaction was stirred overnight at room temperature. The resin was washed with DMF (three times) and a solution of NaBH_3_CN (5 eq) in DMF was added and the resin was stirred at room temperature for 15 min to reduce the Schiff base. In order to release the secondary amine functional group, the resin bound PNA was treated twice with 20% piperidine in DMF for 5 min. The TNBS (Hancock and Battersby, 1976) and chloranil (Blackburn, 2005) tests were used during the synthesis to check for the presence of free N-terminal amine functional groups.

### Purification and characterization of peptide and PNA

The analysis and purification of the crude compounds were carried out on reversed-phase high performance liquid chromatography (RP-HPLC) using Phenomenex Jupiter columns (4.6 × 250 mm, C18, 5 μm) and (21.2 × 150 mm, C18, 5 μm) with solvent A consist of 0.1% TFA in water, while solvent B consist of 0.1% TFA in 100% acetonitrile. All compounds were analyzed and purified by on a 5 μm XB-C18 analytical column (250 Å–4.6 mm, Phenomenex) on Waters system composed of 600-pump controller and 996-diode array detector. Final compounds such as **1**, **1a**, and **2** were purified on a 5 μm XB-C18 preparative column (150 Å–21.2 mm, Phenomenex) before to be tested. Fractions collected from the RP-HPLC were analyzed by matrix-assisted laser desorption/ionization time-of-flight mass spectroscopy (MALDI-TOF MS) on a Bruker Daltonics Autoflex II TOF/TOF. α-cyano-4-hydroxy-cinnamic acid and sinapinic acid were the matrices utilized for characterization.

### *In vitro* cell transfections

The *in vitro* cell-based assay was performed using primary canine myoblasts. All the canine primary cell-based assays were approved by the Institutional Animal Experiment Committees of the National Center of Neurology and Psychiatry. Primary myoblast cells (5 × 10^4^ cells) from CXMD were cultured in growth medium containing DMEM/F-12 1:1 (Invitrogen, San Diego, CA, USA), 20% fetal bovine serum, basic fibroblast growth factor (2.5 ng/ml), and 1% penicillin/streptomycin for 24 h, and then changed to a differentiation medium containing Dulbecco's minimum essential medium with Horse Serum (HS) (2%) and cultured for 3 days. The medium was then changed to a differentiation medium containing a final concentration of 5 μM antisense oligonucleotide (PNA or peptide-conjugated PNA) without transfection reagents, and cultured for 3 days before analyses for RNA. We chose to treat the cells with 5 μM concentration based on our previous studies on PNA and other splice-switching oligonucleotides. This concentration of naked PNA provides a modest level of exon-skipping activity which is not saturating and can be compared to the peptide-PNA constructs.

### Reverse transcriptase polymerase chain reaction

Total RNA was extracted from myoblasts using RNeasy Mini Kit (Qiagen, Hilden, Germany) according to manufacturer's protocol. Then reverse transcriptase polymerase chain reaction (RT-PCR) was performed on 100 ng of total RNA for 35 cycles of amplification using One-Step RT-PCR kit (Qiagen, Chatsworth, CA) following manufacturer's instructions with 0.6 mM of forward primer (exon-5 CTGACTCTTGGTTTGATTTGGA) and reverse primers (exon-10 TGCTTCGGTCTCTGTCAATG). Amplicon detection was carried out with a microchip electrophoresis system in the MultiNA system (Shimadzu, Kyoto, Japan).

## Results and discussion

### On-resin coupling efficiency and removal of Hmb and Dmb

In the first instance, we investigated the on-resin coupling efficiency and removal of Hmb- and Dmb-modified PNA (Offer et al., 1996; Miranda et al., 2000; Abdel-Aal et al., 2014). This allowed us to select the most efficient backbone modification in terms of coupling rates and removal (Scheme S1). The 2-hydroxy-4-methoxybenzaldehyde (Hmb-CHO) or 2,4-dimethoxybenzaldehyde (Dmb-CHO) were introduced at the N-terminal of free amino group of PNA monomer following a two-step protocol with first, the formation of the Schiff base and then reductive amination. For Hmb backbone modification, we synthesized four PNA dimers (AG, CG, GG, TG) starting with guanidine(Bhoc) which is the most bulky PNA monomer and coupling of the next PNA monomer is often difficult due to the steric hindrance. Hmb serves as a functional moiety which enables the subsequent PNA monomer to be readily coupled onto the phenol; the *O*-to-*N* acyl transfer (Tailhades et al., 2015) occurs to form the amide bond and is then removed during the final TFA cleavage. The PNA dimers were analyzed using RP-HPLC and MALDI-TOF mass spectrometry (MS) (Scheme S1A and Table S1). The results showed a full conversion of the monomer into its corresponding dimers that results from the complete O-to-N acyl transfer during the coupling for each of the Fmoc-PNA monomers which is often the limiting step (Lelievre et al., 2016). It is important to note that the phenolic ester (S1) is not stable to the piperidine treatment prior to the final TFA cleavage and consequently, there is no doubt about the formation of the dimers through an amide bond. In the case of Dmb (Scheme S1B), we prepared a library of 16 PNA dimers following a combinatorial chemistry approach (4 B_n_ × 4 B_n+1_). After TFA cleavage, the 16 PNA dimers were analyzed by MALDI-TOF MS to determine if there was any deletion. In all cases, the coupling of the Fmoc-PNA monomer (B_n+1_) had gone to completion as the mass detected was that of the PNA dimer (Table S1). However, an additional ion (+148 Da) was detected when the second PNA monomer (A, C, G, or T) was coupled to the H-(Dmb)-thymine-bound resin. This ion was attributed to the incomplete cleavage of Dmb (+150 Da) that could not be removed despite a second cleavage with higher quantity of scavenger. The incomplete removal of Dmb from thymine was also analyzed by RP-HPLC. The percentage of Dmb removal were 78, 68, 67, and 48% for GT, CT, AT, TT, respectively (Figure S1). In summary, the incomplete removal of Dmb is sequence-dependent but mostly occurs with H-(Dmb)-thymine.

### Effect of Hmb on the synthesis of PNA

**Figure 1:**
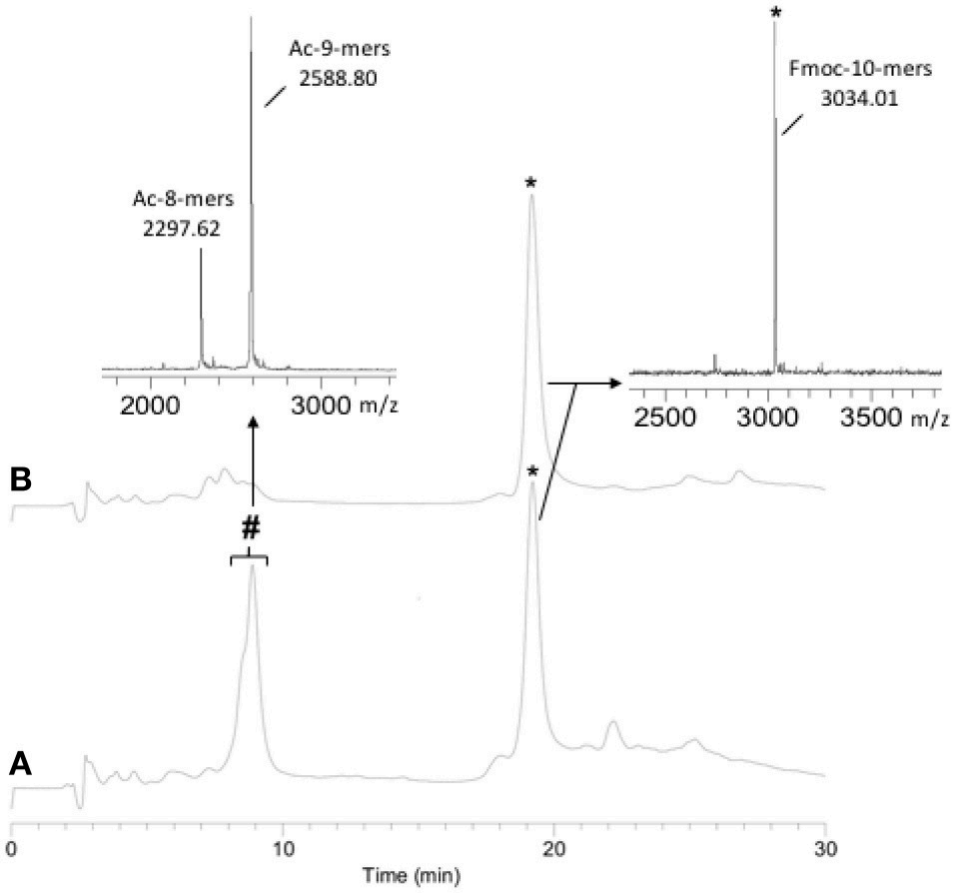
10-mers synthesis without **(A)** and with **(B)** the incorporation of Hmb on G8. **(A)** RP-HPLC: 40% deletion; **(B)** RP-HPLC: no deletion. (^*^) expected PNA: Fmoc-TGGTGAATAG-NH_2_ (10-mers) and (#) deleted PNA: Ac-GTGAATAG-NH_2_ (8-mers) and Ac-GGTGAATAG-NH_2_ (9-mers).

**Scheme 1:**
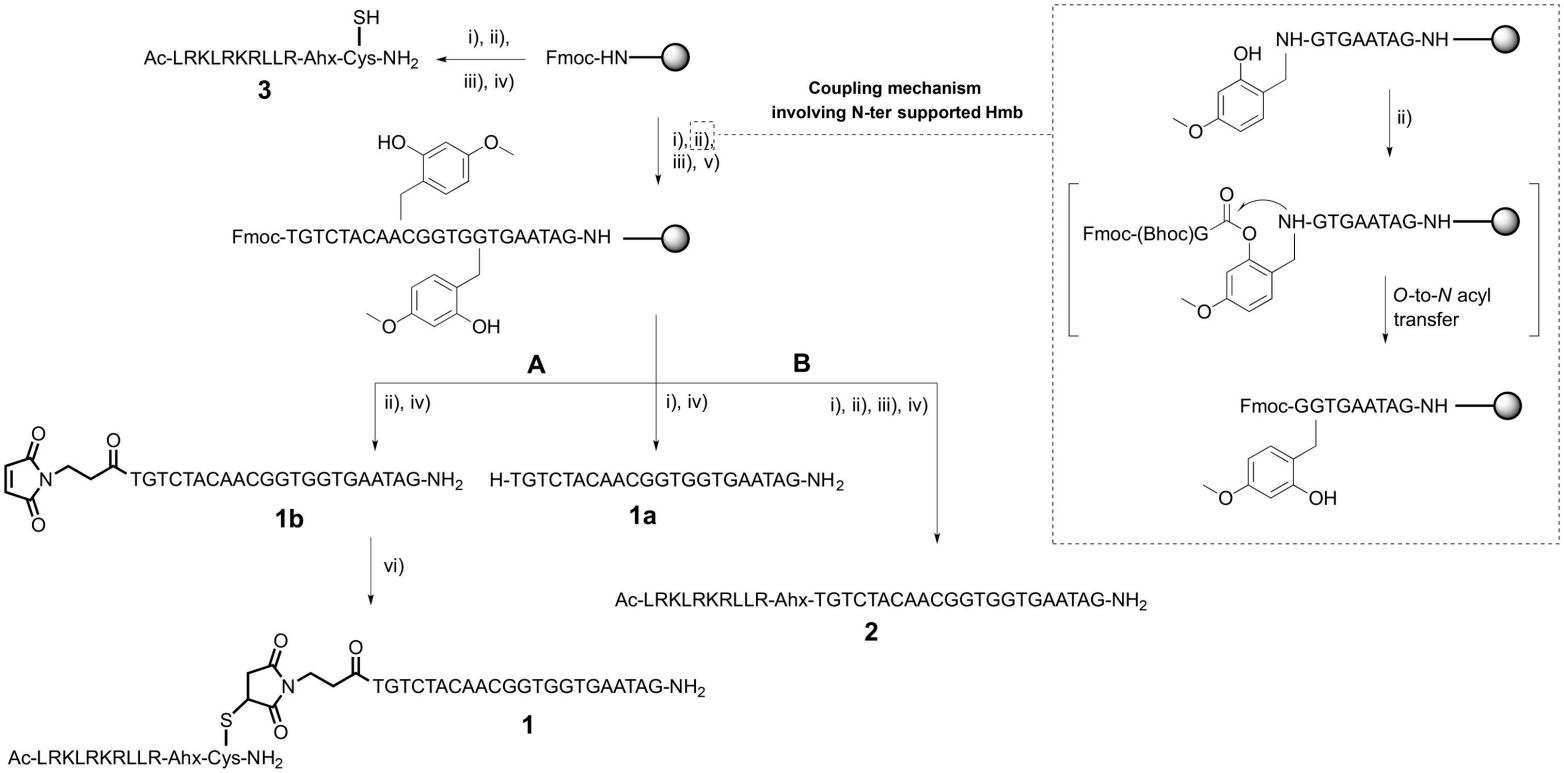
CPP-PNA synthesis: **(A)** thiol-maleimide addition and **(B)** direct solid-phase synthesis. (i) Piperidine, DMF; (ii) Fmoc-AA-OH/Fmoc-PNA monomer, HATU, DIEA, lutidine, NMP or maleimido-propionic acid, DIC, HOBt, DMF for 1b; (iii) Ac_2_O, lutidine, DMF; (iv) TFA, TIS, H_2_O for 1a, 1b, and 2 or TFA, DODT, TIS, H_2_O for 3; (v) Hmb-CHO, AcOH, DMF then NaBH_3_CN, DMF; (vi) 3 and 1b dissolved in PBS then pH was raised to 7.4–7.6 using NH_4_OH solution pH 8.

As the Hmb modification was determined to be superior to Dmb due to its ease of subsequent removal, it was chosen for the synthesis of a 22-mer PNA sequence. The initial synthesis of the PNA was carried out without Hmb backbone modification. The monitoring of the PNA synthesis by RP-HPLC and MALDI-TOF MS after cleavage of an aliquot showed 40% of deleted PNA at the level of the 8-mers and 9-mers (Figure 1A). During RP-HPLC analysis, the retention time difference between the 8, 9, and 10-mers was not clear, hence the 10-mer was cleaved from the resin with the Fmoc group still on in order to distinguish it from truncated 8 and 9-mers. Due to the presence of these major deletions, we chose to incorporate Hmb between G8 and G9 along the sequence to improve the coupling rate of G9 and, consequently, T10 (Scheme 1). As expected, the addition of Hmb on G8 significantly improved the efficiency of G9 coupling and enabled the straightforward synthesis of the 10-mers (Figure 1B).

### Synthesis of long PNA using Hmb backbone modification

**Figure 2:**
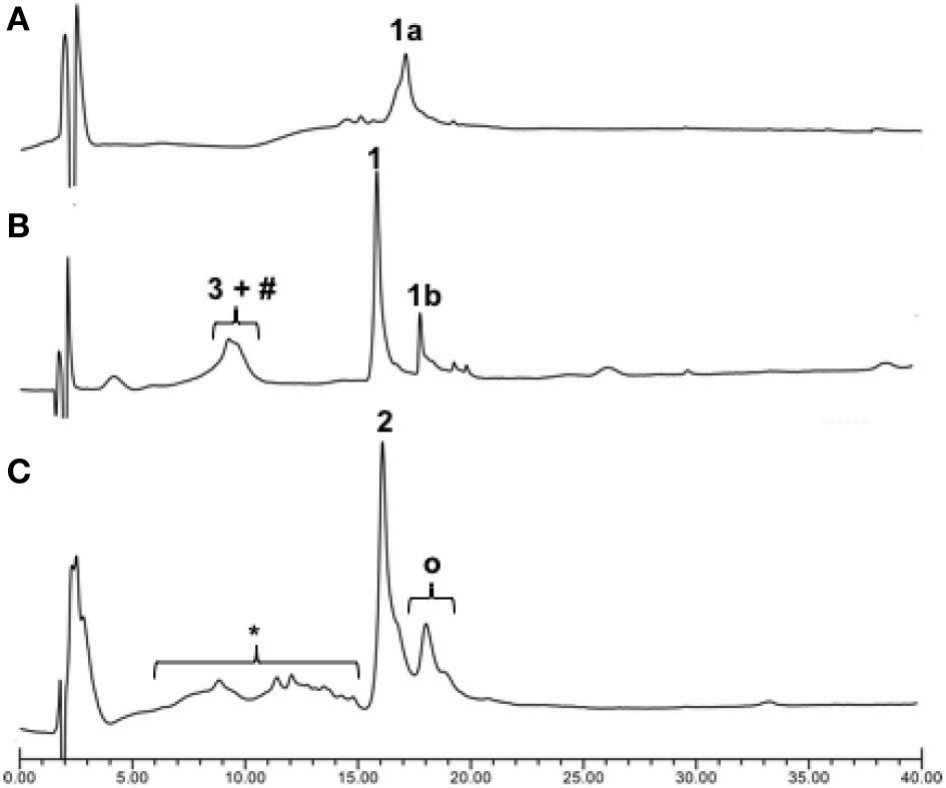
Crude RP-HPLC profiles of PNA/ApoE-PNA. **(A)** Crude PNA 22mers (1a); **(B)** Thiol-maleimide conjugation between 3 (ApoE) and maleimido-PNA (1b) in PBS buffer (pH 7.4) for 30 min and **(C)** the direct SPPS. ^#^Dimeric form of 3, ^*^Deleted CPP-PNA, ^O^Residual Ac-Hmb within the final compound 2.

Once we established the significant beneficial effect of Hmb incorporation on the synthesis of a purine rich 10-mer PNA, the solid-phase assembly was continued with each coupling monitored by the TNBS test. However, the coupling test was unclear after G12 which is often a sign of aggregation and/or difficult coupling. To overcome this problem, a second Hmb was incorporated on A14 prior to the coupling of A15. Following completion of the 22-mer PNA synthesis, it was cleaved from solid phase and analyzed by RP-HPLC (Scheme 1—Compound **1a** and Figure 2A). The RP-HPLC analysis of 22-mer PNA showed a broad peak which is characteristic of long PNAs (Pipkorn et al., 2012). However, as soon as the PNA is conjugated to the cell-penetrating peptides, the RP-HPLC peaks are well-defined and it can be easily purified (Figures 2B,C). Nevertheless, the choice of Hmb backbone modification allows long PNA sequences to be synthesized using the Fmoc/Bhoc strategy. Based on this study, we recommend introduction of Hmb within a purine-rich region such as in our case GTG(Hmb)GTG and –ACA(Hmb)ACG.

### Solid-phase synthesis of peptide-PNA

Since cell-penetrating peptides are often used for intracellular delivery of PNA, we then used two different approaches to synthesize the CPP-PNA (Scheme 1). Firstly, we applied a conventional thiol-maleimide reaction (Figure 2B) between the PNA functionalized with a maleimido-propionic acid at the N-terminus (3′-end) (**1b**) (180 nmol) and a CPP (LRKLRKRLLR-Ahx-C) functionalized with a cysteine at the C-terminus (**3**) to give the construct CPP-PNA (**1**) (23 nmol) in a final yield of 12.8%. In the second approach, we continued the synthesis of ApoE on the same solid-support (Figure 2C). The CPP-PNA (**2**) was obtained in a good purity and yield of 14.4% (28.8 nmol based on 200 nmol of crude) (Figure S2).

### Antisense effect of PNA and ApoE-PNA in differentiated canine myoblast

**Figure 3:**
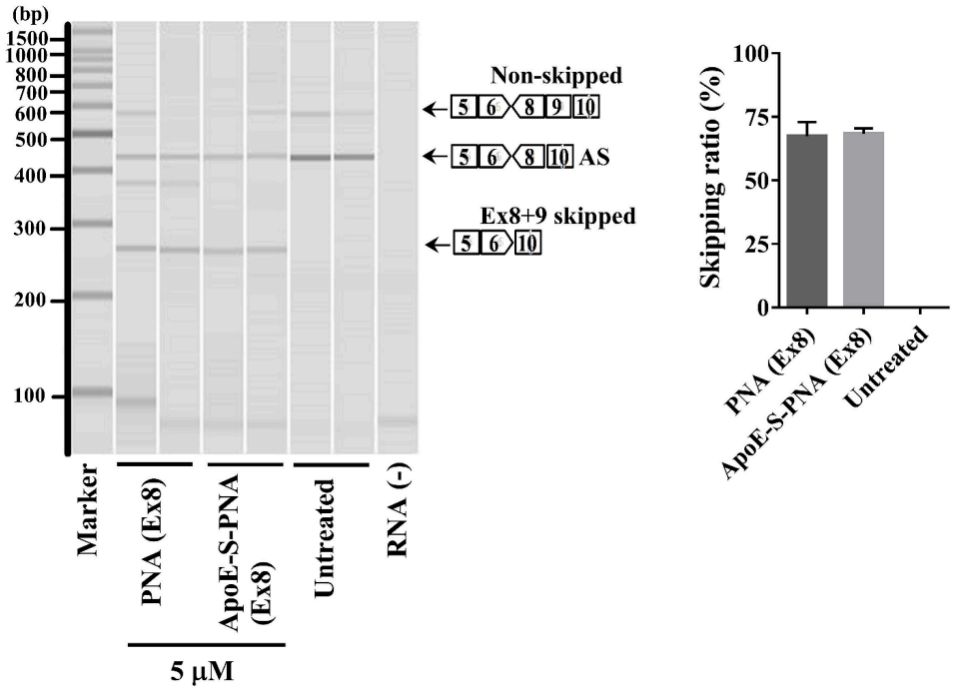
Densitometry analysis of exon-8 skipping in DMD dog myoblasts cells treated with PNA and ApoE-PNA (1) at 5 μM. [AS = alternative spliced. Skipping ratio = skipped band/(non-skipped band + alternative spliced band + skipped band].

The synthesized 22-mer PNA binds to canine dystrophin pre-mRNA and leads to the exclusion of exon-8 in the mature mRNA. Therefore, to confirm the exon-8 skipping activity of the synthesized PNA and ApoE-PNA (**1**), differentiated canine primary myoblasts were treated with PNA or ApoE-conjugated-PNA at 5 μM. Both showed high efficacy of binding to the target dystrophin RNA and skipping exon-8 from both splice variants (non-skipped and alternative skipped AS) (Figure 3). The exon-skipping activity obtained with PNA with or without CPP (ApoE) is almost similar. This can be due to the long treatment time of myoblasts which allows naked PNA to be taken up, albeit slower compared to ApoE-PNA, and produce an exon-skipping effect similar to that of ApoE-PNA.

## Conclusions

In this study, we investigated the effect of Hmb and Dmb backbone modification on the solid-phase synthesis of difficult purine-rich PNA sequences. We have shown for the first time the optimization and successful application of Hmb backbone modification to the synthesis of difficult 22-mer PNA sequence containing ~60% purine bases. This novel application of Hmb also enabled the synthesis of peptide-PNA on the same solid-support in good yield and purity without the need for the separate synthesis of each PNA and peptide.

## Author contributions

JT performed chemical syntheses, experimental design, and drafted the manuscript; HT and YA performed the bioassays; DW, MG, JW, and FS took part in experimental design. All authors worked on the manuscript.

### Conflict of interest statement

DW is employed by the company SpheriTech Ltd. The other authors declare that the research was conducted in the absence of any commercial or financial relationships that could be construed as a potential conflict of interest.
