# Supplementary material for: Solid-Phase Synthesis of Difficult Purine-Rich PNAs through Selective Hmb Incorporation: Application to the Total Synthesis of Cell Penetrating Peptide-PNAs
Source: Front Chem. 2017 Oct 17;5:81. doi: 10.3389/fchem.2017.00081 (PMC5651559; doi:10.3389/fchem.2017.00081)

Supplementary Material

Solid phase synthesis of difficult purine-rich PNAs through selective Hmb incorporation: Application to the total synthesis of peptide-PNAs

J. Tailhades, H. Takizawa, M.J. Gait, D.A. Wellings, J.D. Wade, Y. Aoki and F. Shabanpoor*

*** Address correspondence to:** [fazel.shabanpoor@unimelb.edu.au](mailto:fazel.shabanpoor@unimelb.edu.au)

Scheme S1. PNA dimer library through Hmb (A)/Dmb (B) incorporation. i) Piperidine, DMF; ii) Fmoc-Lys(Boc)-OH or Fmoc-PNA monomer, HATU, DIEA, lutidine, NMP; iii) Hmb-CHO (A) or Dmb-CHO (B), AcOH, DMF; iv) NaBH_3_CN, DMF; v) TFA, TIS, H_2_O.

**Table S1**. Library screening by MALDI-TOF/TOF MS (*m/z* in Da).

| **Starting resin** | **Sequence** | **Theoretical Mass** | **Experimental Mass** | **Additional Mass** |
| --- | --- | --- | --- | --- |
| H-(**Hmb**)G-KK-NH-® | H-**AG**-KK-NH_2_ | 839.44 | 840.25 | - |
|  | H-**CG**-KK-NH_2_ | 815.43 | 816.40 | - |
|  | H-**GG**-KK-NH_2_ | 855.43 | 856.46 | - |
|  | H-**TG**-KK-NH_2_ | 830.43 | 831.00 | - |
| H-(**Dmb**)A-KK-NH-® | H-**AA**-KK-NH_2_ | 823.44 | 824.50 | - |
|  | H-**CA**-KK-NH_2_ | 799.43 | 800.02 | - |
|  | H-**GA**-KK-NH_2_ | 839.44 | 840.22 | - |
|  | H-**TA**-KK-NH_2_ | 814.43 | 815.29 | - |
| H-(**Dmb**)C-KK-NH-® | H-**AC**-KK-NH_2_ | 799.43 | 800.28 | - |
|  | H-**CC**-KK-NH_2_ | 775.42 | 776.07 | - |
|  | H-**GC**-KK-NH_2_ | 815.43 | 816.26 | - |
|  | H-**TC**-KK-NH_2_ | 790.42 | 791.28 | 939.78 |
| H-(**Dmb**)G-KK-NH-® | H-**AG**-KK-NH_2_ | 839.44 | 840.30 | - |
|  | H-**CG**-KK-NH_2_ | 815.43 | 816.19 | - |
|  | H-**GG**-KK-NH_2_ | 855.43 | 856.49 | 1006.80 |
|  | H-**TG**-KK-NH_2_ | 830.43 | 831.32 | - |
| H-(**Dmb**)T-KK-NH-® | H-**AT**-KK-NH_2_ | 814.43 | 814.90 | 962.86 |
|  | H-**CT**-KK-NH_2_ | 790.42 | 791.00 | 939.19 |
|  | H-**GT**-KK-NH_2_ | 830.43 | 831.31 | 979.31 |
|  | H-**TT**-KK-NH_2_ | 805.42 | 806.54 | 954.59 |

**Figure S1**. RP-HPLC analysis of Dmb removal from thymine. A gradient of 0 – 30% acetonitrile (containing 0.1% TFA) in 30 min at a flow rate of 1.5 ml/min was used for the analysis.


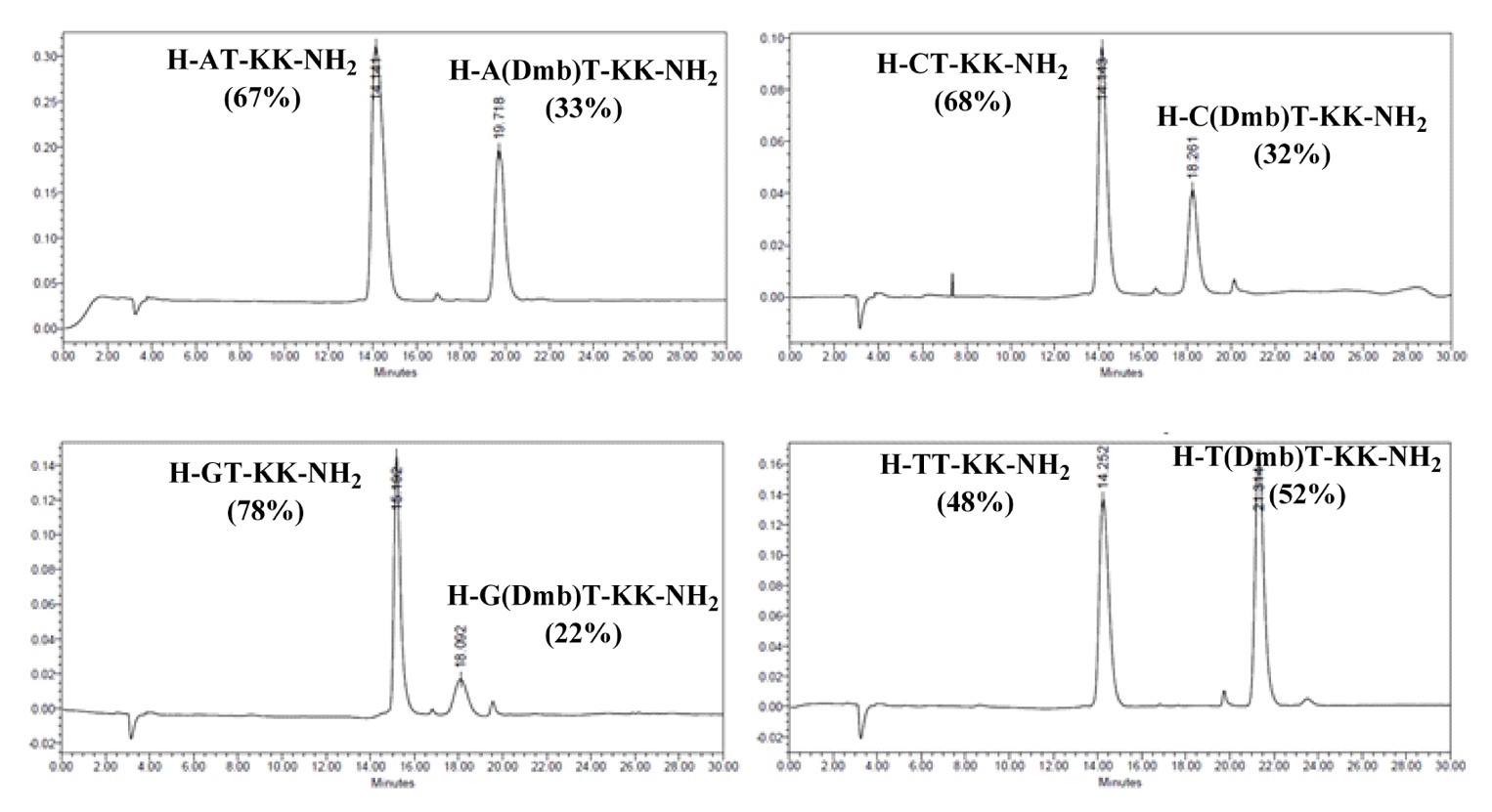
**Figure S2.** RP-HPLC (left) and MALDI-TOF MS (right) analysis of purified compounds **1** and **2**. A gradient of 10 – 40% acetonitrile (containing 0.1% TFA) in 30 min at a flow rate of 1.5 ml/min was used for the analysis of purified ApoE-PNA conjugates. The theoretical mass of conjugates **1** and **2** are 7819.8 and 7547.6 respectively.


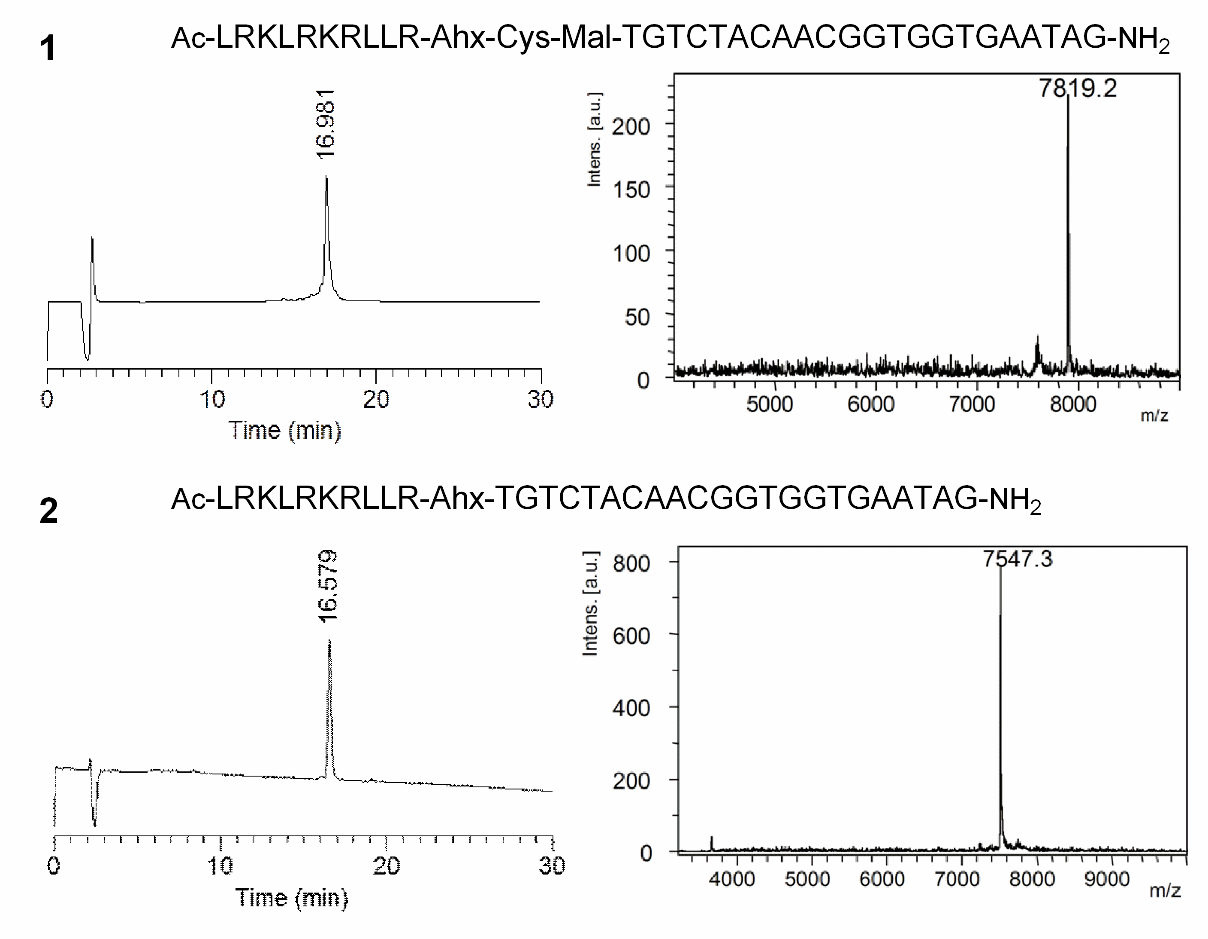

Supplement: Supplementary file 1 [file DataSheet1.docx]
